# Accuracy of peak VO2 assessments in career firefighters

**DOI:** 10.1186/1745-6673-6-25

**Published:** 2011-09-25

**Authors:** Dana C Drew-Nord, Jonathan Myers, Stephen R Nord, Roberta K Oka, OiSaeng Hong, Erika S Froelicher

**Affiliations:** 1 Department of Community Health Systems, School of Nursing, University of California, 2 Koret Way, San Francisco, California 94143, USA; 2 School of Medicine, Stanford University, Palo Alto VA Health Care System, 3801 Miranda Avenue, Palo Alto, California 94304-1290, USA; 3 Premier COMP Medical Group, Inc. 5635 W. Las Positas Blvd., Suite 401, Pleasanton, CA 94588, USA; 4 Palo Alto VA Health Care System, 3801Miranda Avenue, Palo Alto, California 94304-1290, USA; 5 Department of Physiological Nursing, School of Nursing, University of California, 2 Koret Way, San Francisco, California 94143, USA

## Abstract

**Background:**

Sudden cardiac death is the leading cause of on-duty death in United States firefighters. Accurately assessing cardiopulmonary capacity is critical to preventing, or reducing, cardiovascular events in this population.

**Methods:**

A total of 83 male firefighters performed Wellness-Fitness Initiative (WFI) maximal exercise treadmill tests and direct peak VO_2 _assessments to volitional fatigue. Of the 83, 63 completed WFI sub-maximal exercise treadmill tests for comparison to directly measured peak VO_2 _and historical estimations.

**Results:**

Maximal heart rates were overestimated by the traditional 220-age equation by about 5 beats per minute (p < .001). Peak VO_2 _was overestimated by the WFI maximal exercise treadmill and the historical WFI sub-maximal estimation by ~ 1MET and ~ 2 METs, respectively (p < 0.001). The revised 2008 WFI sub-maximal treadmill estimation was found to accurately estimate peak VO_2 _when compared to directly measured peak VO_2_.

**Conclusion:**

Accurate assessment of cardiopulmonary capacity is critical in determining appropriate duty assignments, and identification of potential cardiovascular problems, for firefighters. Estimation of cardiopulmonary fitness improves using the revised 2008 WFI sub-maximal equation.

## Background

Every 23 seconds a fire in the United States requires the services of a career or volunteer fire department [1]. Sudden cardiac death is the most common cause of on-duty death among firefighters and occurs at higher rates than those found in similar occupations, such as police and emergency medical services [2].

A joint task force of the International Association of Firefighters (IAFF) and International Association of Fire Chiefs developed the Fire Service Joint Labor Management Wellness-Fitness Initiative (WFI) in 1997. Revisions in the 1999 and 2008 WFI recognize the firefighter as the "most important asset" in the fire service, and its intent is to improve firefighter function, on-duty effectiveness, and overall quality of life, while reducing morbidity and mortality related to fire fighting [3]. A major component of the WFI is assessment of firefighters' cardiopulmonary capacity, with a stepmill test, sub-maximal, or a maximal exercise treadmill test. The WFI mandates that firefighters have a maximal exercise test at age 40 and every other year thereafter. The maximal exercise test is intended to measure peak VO_2 _(measured as ml/kg^-1^·min^-1^), which is an objective, clinical measure that defines the limits of cardiopulmonary function. Peak VO_2 _reflects an individual's ability to increase their heart rate and stroke volume, and redirect oxygenated blood to muscles for work on demand. Exercising at levels beyond which the cardiopulmonary system can adequately supply oxygen (commonly termed the anaerobic or ventilatory threshold, or VT) involves progressively greater degrees of oxygen-independent muscle metabolism, which is dramatically less efficient than aerobic metabolism, and can compromise cardiovascular function [4].

Quantifying the energy demands of firefighting during fire suppression is difficult due to the inherent dangers of fire suppression tasks. Most efforts to define the arduous physical work demand requirements during firefighting have been focused on establishing the level of metabolic equivalents (METs) (1 MET ≈ 3.5 ml of O_2_/kg/min) using simulated tasks. A MET is a multiple of the resting metabolic rate and is commonly estimated using standardized equations [4]. 10 METs is roughly equivalent to jogging a 10-minute mile; 14 METs is similar to many extended competitive activities such as running or rowing competitively, or bicycle racing at a high level [5]. The estimated METs proposed for firefighting range from 9.6 [6] to 14 [7] (a peak VO_2 _range of 33.6 ml/kg^-1 ^min^-1^·to 49 ml/kg^-1^·min^-1^). Recent analysis of physical aptitude tests among firefighter recruits demonstrated that male recruits' average VO_2 _requirement was 38.5 ml/kg^-1^·min^-1 ^(11 METs) to complete a timed simulated firefighting assessment course [8]. Measurement of functional capacity in 23 firefighters suggested that a mean of 41.54 ml/kg^-1^·min^-1 ^(11.9 METs) is required to complete standard fire suppression tasks while wearing personal protective equipment [9].

Firefighting work demands can be extreme and accurate assessment of cardiopulmonary status, as well as detection and treatment of any underlying cardiovascular disease, is critical to insure firefighter fitness for duty and prevent on-duty cardiac events or death. The 1999 WFI sub-maximal exercise test was found to overestimate true peak VO_2 _in individual firefighters [10]. Concern about overestimation led to a revised equation for estimating peak VO_2 _from sub-maximal exercise treadmill tests in the 2008 WFI.

## Materials and methods

Given that previous sub-maximal exercise test results in the WFI were shown to overestimate peak VO_2_, and that the WFI maximal exercise treadmill protocol has not been validated for accuracy in the literature, this study was undertaken to assess the validity of both the maximal and revised sub-maximal exercise treadmill peak VO_2 _estimates in firefighters. Specifically, the present study tested the following comparisons: (a) estimated maximal heart rate (220 - age) to actual measured maximal heart rate; (b) WFI maximal exercise estimated peak VO_2 _to directly measured peak VO_2_; c) averaged pre-revision sub-maximal estimated peak VO_2 _to revised sub-maximal estimated peak VO_2_; and (d) directly measured peak VO_2 _to revised WFI sub-maximal estimated peak VO_2_.

### Study Setting and Participants

The study setting was a medium-sized suburban fire department in the eastern region of the San Francisco Bay Area in northern California. This department serves approximately 163,000 citizens and covers 46 square miles. All firefighters (*N *= 105) assigned to suppression duties were recruited, including firefighters, firefighter/paramedics, firefighter/engineers, firefighter/captains and battalion chiefs. There were no women suppression firefighters in the department studied. This is consistent with national career firefighter statistics as women only represent approximately 4.5% of the fire service [11]. All testing took place during a five-week period between December 2008 and January 2009.

Inclusion criteria for participation required that each participant had successfully completed a WFI examination within the previous nine months and achieved a minimum of 10 METs (peak VO_2 _of 35 ml/kg/min), on either a sub-maximal (using the pre-2008 equation), or maximal exercise treadmill test. Exclusion criteria included injury, illness, or scheduling conflicts that precluded testing during the study period. The final study population consisted of 83 male career firefighters from all suppression ranks in this department.

The study was conducted with approval of the University of California San Francisco Committee on Human Research. Signed informed consents were obtained and all testing was conducted during on-duty hours with the approval of the department and union local.

Testing occurred at an occupational health clinic where previous WFI examinations for this fire department had been conducted. A physician board certified in internal medicine and occupational medicine, and a nurse practitioner experienced in exercise testing, performed all treadmills and direct VO_2 _measurements. Participants arrived on the day of scheduled testing with their assigned duty crew, with gym clothes and running shoes appropriate for completing a maximal exercise test.

### Measurements

Data collection consisted of medical record abstraction for demographics, cardiovascular risk factors and exercise test information. Demographic characteristics included age, rank, and years of fire service. Definitions of cardiovascular risk factors were obtained from the American Heart Association, Adult Treatment Panel III (ATP III), The Seventh Report of the Joint National Committee on Prevention, Detection, Evaluation and Treatment of High Blood Pressure (JNC7), and the Centers for Disease Control and Prevention [12-15]. All serum samples were analyzed at the same hospital-based certified laboratory (Centers for Medicare and Medicaid Services Clinical Laboratory Improvements Amendments (CLIA)). Cardiovascular risk factors of the participants are summarized in Table 1.

**Table 1:** Participant Cardiovascular Risk Factor Profile - Maximal Exercise Treadmill-Peak VO_2 _Assessment (n = 83)

Risk Factor	Mean, SD
**Body Mass Index (kg/m^2^)**	28.2 (± 3.9)
**Systolic BP**	117 (± 10)
**Diastolic BP**	69 (± 7)
Total Cholesterol* (mg/dL)	197 (± 38)
HDL** (mg/dL)	47 (± 11)
LDL** (mg/dL)	126 (± 36)
**Cholesterol/HDL Ratio**	4.35 (± 1.17)
**Triglycerides (mg/dL)**	118 (± 70)

*-fasting

**- HDL - high density lipoprotein; LDL - low density lipoprotein

#### Maximal Heart Rates

Maximal estimated heart rates were calculated as 220-age. Directly measured maximal heart rates were determined from the electrocardiogram at the point of volitional fatigue as determined by the firefighter and corroborated by the direct VO_2 _assessment indicating that they had crossed the VT.

#### Maximal Exercise Treadmill with Direct Peak VO_2 _Assessment

All 83 participants completed a maximal exercise test using the 2008 WFI Protocol with concurrent direct peak VO_2 _measurements. Maximal exercise treadmill tests were considered complete when the firefighter indicated volitional fatigue (*n *= 83, see above), or if terminated by the testing physician due to concerns about cardiopulmonary distress (*n *= 0). The WFI protocol is a modified ramp protocol comprised of a 3-minute warm-up period at 3 mph - 0% grade, followed by fifteen 1-minute stages. Stage 1 begins at 4.5 mph and 0% grade, with the treadmill incline increasing 2% and speed increasing by 0.5 mph alternately in stages 2 through 15. The WFI maximal exercise treadmill estimates peak VO_2 _based on the American College of Sports Medicine metabolic equation for running [16].

Peak VO_2 _was obtained using the Cardio Coach CO_2_™ VO_2 _Fitness Assessment System, Model 9001-RMR (Korr Medical Technologies, Salt Lake City, Utah). The Cardio Coach CO_2_™ is an economical, portable metabolic testing device that is feasible for use in a clinic and has been previously validated for measurement of peak VO_2 _levels [17,18]. The Cardio Coach CO_2_^TM is ^a dual gas analyzer (O_2 _and CO_2_) that automatically calibrates to standard temperature and pressure, dry at the beginning of each testing cycle. The Cardio Coach CO_2_™ measures heart rate using the Polar T-31 heart monitor (Polar, Inc., Lake Success, NY). Heart rate and VO_2 _(ml/kg^-1^· min^-1^), VCO_2 _(ml/kg^-1^· min^-1^), VE/VO_2, _VE/VCO_2_, VE in L/min, FeO_2_%, Fe CO_2_%, and respiratory exchange ratio are graphically reported every 15 seconds. The Cardio Coach CO_2_™ uses the ventilatory equivalents method (Ve/VO_2_) to detect VT (Korr Medical Technologies, 2009).

#### Revised Sub-maximal Exercise Treadmill Assessments

In the latter part of 2008, the WFI introduced a revised equation for estimating peak VO_2_: peak VO_2 _= 56.981 + (1.242 × TT) - (0.805 × BMI), where TT is the test time required to achieve target heart rate, and BMI is Body Mass Index. The 2008 WFI calculates target sub-maximal heart rate (208 - (0.7 × age) × 0.85, whereas previous sub-maximal heart rates were based on (220-age) × 0.85 [3,19].

Of the 83 firefighters who volunteered for the maximal exercise treadmill tests and directly measured peak VO_2_, 63 subsequently completely their annually scheduled WFI examination, which included a sub-maximal exercise treadmill test, within the subsequent four to eight weeks. These subsequent WFI sub-maximal exercise treadmill tests, using the revised equation, took place under identical conditions as the study WFI maximal exercise treadmill tests but without the direct VO_2 _measurement. The sub-maximal test uses the WFI treadmill protocol (see above) but terminates 15 seconds after the firefighter reaches their target heart rate.

#### Pre-revision Sub-maximal Exercise Treadmill Assessments

Prior to the 2008 WFI revision there was no published equation for the estimation of peak VO_2 _from the sub-maximal exercise treadmill. The estimated peak VO_2 _was determined by duration of the test and stage achieved [19]. Between one and seven historical sub-maximal test results were available for each of the 63 participants, and were averaged to create comparative historical variables.

### Procedure

Participant's height, weight and resting blood pressure was measured. A resting electrocardiogram (ECG) was completed, using the Welch-Allyn Schiller AT-10 6-Channel electrocardiograph/treadmill (San Diego, California). Upon completion of the resting ECG the Mason-Likar lead configuration was modified to accommodate the exercise treadmill [4]. The participant was then fitted with the appropriate 2-way non-rebreathable mask (Hans-Rudolph, Inc., Shawnee, Kansas). The mask completely covered the nose and mouth of the participant and was checked for air leaks to eliminate extraneous room air from affecting the interpretation of peak VO_2_. A standing electrocardiogram was obtained and the treadmill was initiated. At test termination the firefighter recovered in the supine position. Available data from the maximal exercise treadmills is detailed in Table 2.

**Table 2:** Maximal Exercise Treadmill Data (n = 83)

	Minimum	Maximum	Mean, SD
**Resting Systolic**	102	164	122 (±10)
**Resting Diastolic**	60	100	73 (±8)
**Resting Heart Rate**	42	91	63 (±10)
**Maximal Heart Rate**	130	194	174 (±10)
**Peak VO_2 _Actual**	26.3	69.5	43.6 (±9.1)
RER* - Peak Exercise	0.90	1.28	1.09 (± .07)

* - Respiratory Exchange Ratio

### Statistical Analyses

Prior to all analysis all data were examined using stem and leaf plots and found to have normal distribution. Dependent *t*-tests were conducted on all 83 participants to test for differences between:

1) Estimated maximal heart rate (220 - age) and directly measured maximal heart rate.

2) WFI maximal exercise treadmill estimated peak VO_2 _and directly measured peak VO_2_.

Additional dependent *t*-tests were conducted on the results of the 63 participants who subsequently performed a revised WFI sub-maximal exercise treadmill test for differences between:

1) Averaged pre-revision WFI sub-maximal exercise treadmill estimated peak VO_2 _mean (converted to METs) to revised WFI sub-maximal exercise treadmill estimated peak VO_2 _(converted to METs).

2) Directly measured peak VO_2 _(converted to METs) to revised WFI sub-maximal exercise treadmill estimated peak VO_2 _(converted to METs).

All dependent *t*-tests were two tailed, with α = 0.05 used for statistical significance. Statistical analyses were performed using SPSS Version 15.0 (SPSS, Inc., Chicago, Illinois).

## Results

There were 105 active suppression male career firefighters eligible for participation in the study. Of those, five were new hires who had not completed a WFI examination. Six firefighters chose not to participate; of the 94 choosing to participate 11 could not be scheduled for maximal exercise tests due to injury, illness or scheduling conflicts resulting in an *n *= 83 for this study. The participants' ages ranged from 26 to 57 years with a mean of 41.1; 94% of the participants were Caucasian, and 6% were Hispanic or African-American. The years of firefighting ranged from 2 to 34 with a mean of 15.6.

### Maximal Estimates and Measurements

The traditional maximal heart rate estimation (220 - age) was significantly higher than measured maximal heart rate (178.6 vs. 173.6 with a mean difference of 4.96 beats/min, p < 0.001, 95% CI: 3.03, 6.90). Estimated peak VO_2 _was significantly higher than directly measured peak VO_2 _(47.7 vs. 43.6, with a mean difference of 4.06 ml/kg/min, (1.16 METs) p < 0.001, 95% CI: 2.88, 5.23).

### Sub-maximal Estimates and Measurements

Within four to eight weeks of the maximal exercise treadmill tests 63 participants completed a sub-maximal exercise treadmill test (using the revised 2008 WFI equation). Their average age was 40.19 years (± 6.9) and average years of firefighting was 14.4 (± 6.8). All firefighter suppression ranks were represented in this sub-group. The subsequent examination allowed for comparison of the revised sub-maximal exercise treadmill peak VO_2 _estimate to an averaged pre-revision (comparative historical variable) sub-maximal exercise treadmill peak VO_2 _estimate and the recently obtained directly measured peak VO_2_. For simplicity in reporting sub-maximal results all peak VO_2 _results were converted to METs (peak VO_2_/3.5).

A statistically significant difference was found between pre-revision sub maximal exercise treadmill peak METs mean estimates and revised sub-maximal peak METs estimates (14.81 vs. 12.58, with a mean difference of 2.23 METs, p < 0.001, 95% CI: 1.86, 2.59) These findings support previous research determining that WFI sub-maximal peak METs estimates prior to the 2008 revision were overestimated [10]. Revised sub-maximal treadmill METs estimates did not differ from directly measured maximal exercise treadmill METs, indicating that the revised 2008 estimating equation is a reasonable estimate of METs (12.64 vs. 12.58 with a mean difference of .07 METs, *p *≤ .76, 95% CI: -.39, .54) This represents additional validation of the accuracy of the new estimating equation [3]. All maximal and sub-maximal comparisons are summarized in Table 3.

**Table 3:** Comparisons: Heart Rate, Peak VO_2_, Estimated METs

	*n *	Mean	SD	SEM	95% CI Lower	95% CI Upper	t	d	Sig(2-tailed)
Estimated Max.									
HR: Actual Max.	83	4.96	8.87	.97	3.03	6.9	5.09	82	.00
HR									
Estimated peak VO_2_: Direct measure peak VO_2_	83	4.06	5.39	.59	2.88	5.23	6.85	82	.00
Pre-revision METs									
Est.: Revised METs estimate	63	2.23	1.46	.18	1.86	2.59	12.14	62	.00
Direct METs:									
Revised Sub-maximal METs estimate	63	.07	1.85	.23	-.39	.54	.31	62	.76

## Discussion

Fire departments often struggle to determine fitness for duty for their members who return from an injury or illness, prepare to embark on wildland strike teams, heavy rescue missions, or for daily work assignments. There are ongoing efforts to define minimally acceptable and safe fitness levels; levels that should be informed by the energy requirements needed during a firefighter's tour of duty. Maximum directly measured METs for the firefighters in this study ranged from 7.5 to 19.9, indicating that some participants might have a difficult time meeting the demands of the job while others appear adequately fit. Four different methods of cardiopulmonary assessment are compared here: direct measurement of peak VO_2_, estimated peak VO_2 _derived from a maximal exercise treadmill equation, historical average of pre-revision estimated peak VO_2 _sub-maximal exercise treadmills, and estimated peak VO_2 _derived from the revised (2008) sub-maximal exercise treadmill equation. Directly measured peak VO_2 _is the most objective and considered the "gold standard" of the four methods [4].

The difference observed in maximum heart rate between directly measured maximum heart rate (while wearing a non-rebreathable mask), and a 220-age estimated maximum heart rate (part of the maximal exercise treadmill estimation equation) provides some explanation for the over-estimation. Estimated maximal heart rates were about 5 beats per minute higher than those measured during peak exercise. Heart rates are a method used on the fire ground to evaluate a firefighters' capability to re-enter the fire scene. Using target heart rates that exceed true maximums, or percentages of estimated maximum heart rates that are inaccurate, could result in dangerous duty assignments.

Assessment of direct peak VO_2 _and maximal exercise treadmill results indicate that the equation utilized by the WFI maximal treadmill over-estimates peak VO_2 _by an average of 4.06 ml/kg^-1 ^·min^-1^, or approximately 1 MET. If a firefighter's fitness level is less than optimal, or if they have underlying cardiovascular disease, this overestimation could lead to on-duty clearances that could prove compromising.

Revised sub-maximal exercise treadmill peak VO2 estimates were compared to averaged pre-revision historical sub-maximal exercise peak VO_2 _estimates. The average overestimation of the historical mean was approximately 2 METs. This finding supports the Mier and Gibson report (2004) that the pre-revision WFI sub-maximal treadmill equation overestimated peak VO_2_, and that those equation results should be used with caution for duty assignment decisions.

The comparison of directly measured peak VO_2 _to the revised sub-maximal exercise treadmill peak VO_2 _estimates (*n *= 63) found that there were no differences between the two assessment methods. When comparing revised WFI sub-maximal exercise treadmill peak VO_2 _estimates to previous years of testing, or to reports in the literature, careful consideration must be given to which estimation method was used. The same task, measured with different estimating equations, can result in different results as demonstrated herein.

### Limitations and Strengths

The limitations of our study include the self-selection bias of the participants, the limited gender and ethnic demographics of the group (all male, predominantly Caucasian), and the range in number of historical sub-maximal exercise treadmill VO_2 _estimates, resulting in a less than ideal comparison group. While testing was completed within a four month period, it included the winter holiday season which may have had a seasonal influence on fitness behavior (resulting in an increase or decrease in exercise intensity). The composition of the sample is reflective of the department in terms of gender and ethnicity. There is an average four to eight week gap between the direct measure peak VO_2 _and the sub-maximal exercise treadmill peak VO_2 _assessment without any documentation of fitness behaviors. However, any fitness improvement on the part of firefighters in the interim would have directed the results towards the null.

The strengths of our study include the number of participants, their range in age, rank, firefighting experience, and their experience with the WFI protocol. The availability of seven years historical data can be viewed as a strength. Use of the mask to measure peak VO_2 _was familiar to the participants as they routinely work with self-contained breathing apparatus. The ability to perform all testing components while on duty encouraged participation. There were no incentives offered for participation. All testing was completed in the same facility using the same equipment and personnel, thus increasing consistency of testing and inter-rater reliability.

### Clinical Implications

Firefighters who have been tested using earlier estimation equations may require careful explanation as to a noticeable drop in test results when using the revised 2008 WFI equation. Participants are likely to be disappointed to see a reduction in their "fitness level" when they have not changed their patterns, nor workout habits, between testing cycles. Again, if a fire fighter falls into the lower fitness categories, or has underlying cardiovascular disease, inaccurate estimates could contribute to cardiac compromise.

## Conclusions

In order to protect firefighters from potentially life-threatening cardiac situations it is imperative that exercise testing results are accurate, whether the test is being used for duty assignment or part of a comprehensive risk assessment. The results from the revised sub-maximal exercise treadmill estimation equation appear to accurately reflect directly measured peak VO_2 _results. WFI maximal treadmill peak VO_2 _estimates should be interpreted with caution, especially as they appear to over-estimate METs by an average of 1. Given the potential for over-estimation of fitness, providers who make fitness-for-duty assessments should consider the energy requirements of the job, any underlying cardiovascular risk factors, and the method of testing used when recommending return to, or continuation of, duties. These findings support the continuation and further expansion of reliable exercise testing of firefighters, within the context of a cardiovascular disease prevention program such as the WFI.

Performing measured peak VO_2 _and maximal exercise treadmill tests can be challenging for fire departments to accomplish due to limited resources. The 2008 WFI sub-maximal exercise treadmill test can be safely administered outside of a medical setting using tools that are often available within the fire department (treadmill, stopwatch, and Polar heart monitor). Disadvantages of the sub-maximal treadmill test are the limited means for assessing underlying cardiovascular conditions, and the inability to determine maximal cardiovascular performance directly. However, the revised 2008 sub-maximal treadmill peak VO_2 _estimation equation is a valid tool to assess interim progress in cardiovascular training programs.

## Competing interests

Dr. Drew-Nord and Dr. Nord own the occupational medicine practice where this research was conducted and contract with various fire agencies to provide WFI services. This relationship was determined to represent no conflict of interest by the Institutional Review Board of the University of California, San Francisco. The remaining authors declare that they have no competing interests.

## Authors' contributions

All of the authors contributed substantially to the conception, design, data acquisition and analysis, manuscript drafts and revisions of this study. Each has given final approval for publication.

## References

[B1] Firefighter Statistics http://www.usfa.dhs.gov/statistics/estimates/nfpa/index.shtm retreived August 3, 2011

[B2] United States Fire Administration Corporation T: Firefighter Fatality Retrospective Study Firefighter Fatality Retrospective Study, April 2002/FA-220 2006

[B3] Fire Service Joint Labor Management Wellness-Fitness Initiative http://www.iaff.org/HS/Well/wellness.html

[B4] Froelicher VF Myers J Exercise and the heart 2006 5 Philadelphia: Saunders Elsevier

[B5] Fletcher GF Froelicher VF Hartley LH Haskell WL Pollock ML Exercise standards. A statement for health professionals from the American Heart Association Circulation 1990 82 2286 2322 10.1161/01.CIR.82.6.2286 2242557

[B6] Sothmann MS Saupe K Jasenof D Blaney J Heart rate response of firefighters to actual emergencies. Implications for cardiorespiratory fitness J Occup Med 1992 34 8 797 800 10.1097/00043764-199208000-00014 1506937

[B7] Malley KS Goldstein AM Aldrich TK Kelly KJ Weiden M Coplan N Karwa ML Prezant DJ Effects of fire fighting uniform (modern, modified modern, and traditional) design changes on exercise duration in New York City Firefighters J Occup Environ Med 1999 41 12 1104 1115 10.1097/00043764-199912000-00015 10609231

[B8] Williams-Bell FM Villar R Sharratt M Hughson RL Physiological demands of the firefighter candidate physical ability test Medicine and Science in Sports and Exercise 2009 41 653 662 10.1249/MSS.0b013e31818ad117 19204584

[B9] Adams J Roberts J Simms K Cheng D Hartman J Bartlett C Measurement of functional capacity requirements to aid in the development of an occupation-specific rehabilitation training program to help firefighters with cardiac disease safely return to work American Journal of Cardiology 2009 103 762 765 10.1016/j.amjcard.2008.11.032 19268728

[B10] Mier CM Gibson AL Evaluation of a treadmill test for predicting the aerobic capacity of firefighters Occup Med (Lond) 2004 54 6 373 378 10.1093/occmed/kqh008 15347781

[B11] Women in the fire service http://www.nfpa.org/itemDetail.asp?categoryID=955&itemID=23601&URL=Research/Fire%20statistics/The%20U.S.%20fire%20service&cookie%5Ftest=1 retrieved August 3, 2011

[B12] Men and cardiovascular disease risk factors http://www.heart.org/HEARTORG/Conditions/HeartAttack/UnderstandYourRiskofHeartAttack/Understand-Your-Risk-of-Heart-Attack_UCM_002040_Article.jsp retrieved August 3, 2011

[B13] Healthy weight - it's not a diet, it's a lifestyle! http://www.cdc.gov/healthyweight/assessing/bmi/adult_bmi/index.html#Interpreted

[B14] Third report of the expert panel on detection, evaluation, and treatment of high blood cholesterol in adults (ATP III) http://hp2010.nhlbihin.net/atpiii/calculator.asp?usertype=prof 7828323

[B15] National Institute of Health National Heart Lung and Blood Institute JNC7 Guidelines 2006

[B16] American College of Sports Medicine Guidelines for exercise testing and prescription 2000 6 Baltimore: Lippincott, Williams & Wilkins

[B17] Jensky NE Vallejo AF Ong MD Schroeder ET Validation of the Cardio Coach for sub-maximal and maximal metabolic exercise testing Medicine and Science in Sports and Exercise 2005 37 5 S231

[B18] Dieli-Conwright CM Jensky NE Battaglia GM McCauley SA Schroeder ET Validation of the CardioCoach CO2 for submaximal and maximal metabolic exercise testing The Journal of Strength and Conditioning Research 2009 23 1316 1320 10.1519/JSC.0b013e3181a3c5e8 19528838

[B19] International Association of Fire Fighters The Fire Service Joint Labor Management Wellness-Fitness Initiative 1999 2 Washington, D.C.: International Association of Fire Fighters

